# Overexpression of Oct4 in porcine ovarian stem/stromal cells enhances differentiation of oocyte-like cells in vitro and ovarian follicular formation in vivo

**DOI:** 10.1186/s13048-016-0233-z

**Published:** 2016-04-12

**Authors:** Yeon-Mi Lee, Tae-Ho Kim, Jeong-Hyeon Lee, Won-Jae Lee, Ryoung-Hoon Jeon, Si-Jung Jang, Sun-A Ock, Sung-Lim Lee, Bong-Wook Park, Gyu-Jin Rho

**Affiliations:** Department of Theriogenology and Biotechnology, College of Veterinary Medicine, Gyeongsang National University, 501, Jinju-daero, Jinju, 660-701 Republic of Korea; Animal Biotechnology Division, National Institute of Animal Science, Rural Development Administration, Suwon, 441-706 Republic of Korea; Department of Oral and Maxillofacial Surgery, School of Medicine and Institute of Health Science, Gyeongsang National University, Jinju, 660-702 Republic of Korea; Research Institute of Life Sciences, Gyeongsang National University, 501, Jinju-daero, Jinju, 660-701 Republic of Korea

**Keywords:** Oocyte-like cells, Folliculogenesis, Oct4-overexpression, Estradiol, FSH, Porcine

## Abstract

**Background:**

Recent findings have revealed that the female gonad may have regenerative activity with having germ line stem cells in juveniles and adults. Application of these germ line stem cells could be an alternative therapy for reproductive disorders in regenerative medicine.

**Methods:**

To enhance the potency of differentiation into oocyte-like cells (OLCs) and folliculogenesis, we overexpressed Oct4 in ovarian stem/stromal cell (OvSCs) and examined the cellular properties related to stemness and self-renewal ability and finally demonstrated the ability of in vitro differentiation and folliculogenesis.

**Results:**

Ovarian cortex included putative stem cells in terms of AP activity, cell cycle status, cell proliferation, expression of mesenchymal lineage surface markers and pluripotent transcriptional markers. Further, Oct4 transfected OvSCs (Oct4-OvSCs) were enhanced their AP activity and cell proliferation compared to OvSCs. The potential on in vitro differentiation into OLCs and in vivo folliculogenesis was also evaluated in OvSCs and Oct4-OvSCs, respectively. Oct4-OvSCs possessed higher oogenesis potential in vitro than OvSCs, in terms of expression of germ cell markers by RT-PCR and the number of OLCs. When OvSCs and Oct4-OvSCs were xeno-transplanted into infertile mice ovaries, the OvSCs transplantation induced new primary follicle formation and hormonal levels of estradiol and FSH remained similar to that of normal mice. However, Oct4-OvSCs possessed higher ability for folliculogenesis based on inducing developing follicles with thecal layer and granulosa cells and more similar estradiol level to normal mice.

**Conclusions:**

These findings demonstrated that putative stem cells were present in ovarian cortex and exhibited differentiation ability into OLCs and folliculogenesis in vivo, and Oct4-overexpression enhanced these ability, suggesting their cellular models based on gene therapy in understanding the mechanisms of oogenesis and folliculogenesis, and finally in view of reproductive cell therapy.

## Background

The prevailing belief was that all oocytes in mammals originate from the fetal period of life and persist, but decrease in their number through the reproductive phase of females. However, the recent findings have shown that the female gonad of juveniles and adults may have regenerative activity and possess germ line stem cells, which can be considered as an alternative regenerative medicine therapy for reproductive disorders.

Germline stem cells derived from the mouse ovaries gave rise to oocytes and follicles [1, 2], and following ovarian transplantation, the cells completed oogenesis and mating resulted in the birth of offspring [3]. In addition, oocytes-like cells (OLCs) were generated in vitro using stem cells derived from extra-gonadal origins, including bone marrow and peripheral blood [4], skin [5–7], pancreas [8] and adipose tissue [9]. Based on the above findings, multipotent stem cells might possess the ability to generate OLCs under specific culture conditions.

It has been reported that the ovarian stem/stromal cells (OvSCs) are undifferentiated, self-renewal with multi-lineage differentiation capacity into adipocytes, osteocytes, chondrocytes and neurons in vitro [9]. Similar to mesenchymal stem cells (MSCs), which shows ability for self-renewal and differentiation into multiple mesenchymal lineages in vitro [10], OvSCs also known to express Oct4, Sox2 and Nanog, which are considered to be an important early transcription factors responsible for the regulation of stem cells pluripotency, and revealed the expression of mesenchymal lineage-cell surface markers such as CD44, CD29 and CD90 [11]. However, the evaluation of germ cell specific markers [growth differentiation factor 9b (GDF9b), C-Mos, deleted in azoospermia-like (DAZL), Vasa, zona pellucida C (ZPC), Stella and c-kit] and folliculogenesis marker [follicular stimulating hormone receptor (FSHR)] expression is an important criteria for characterizing OLCs in addition to the assessment of pluripotent transcriptional marker (Oct4) expression [12–14].

In humans, it has been reported that the germ line stem cells exist in ovaries after birth and had the potency for oogenesis in vitro and in vivo. The occurrence of putative ovarian stem cells with no primordial germ cell characteristics which develop into OLCs and blastocyst-like structures was also reported in the ovarian surface epithelium [15]. A recent study has demonstrated that the ovaries of reproductive-aged women possess oogonial stem cells, and these cells were not only being propagated in vitro, but they also generated oocytes in vitro and in vivo [16]. Additionally, a new technology has also been developed to generate transgenic offspring’s by transplantation of female germ cells, transfected with exogenous genes, directly into ovaries [3, 17]. Previously it has been demonstrated that the injection of human germline stem cells isolated from the ovarian cortical biopsies lead to the formation of oocytes after xeno-transplantation into mice [16]. However, additional studies are required in large animal model such as pig, which has both physiological and genetical characteristics similar to humans, rather than relying solely on mice model to understand the mechanism of oogenesis.

Numerous genes are involved in regulating the differentiation ability of stem cells into germ cells [5, 18, 19]. Further, overexpression of Vasa in human embryonic stem cells (ESCs) and induced pluripotent stem cells (iPSCs) has promoted the meiotic progression of germ cells in vitro [20].

Oct4, a regulator of pluripotency [11, 21, 22] expressed in pluripotent stem cells, can induce pluripotency in somatic cells upon transfection [22]. Nevertheless, Oct4 is also expressed in germ cells, which retain the pluripotency through embryogenesis [11]. Recent studies have shown that germline stem cells derived from ovaries also possessed pluripotency as evidenced by the expression of Oct4 [2, 3]. However, no study has been investigated whether the overexpression of Oct4 affects the differentiation potential of ovarian stem/stromal cells (OvSCs) into female germ cells. Therefore, we hypothesized that the overexpression of Oct4 in undifferentiated OvSCs is liable to increase the efficiency of differentiation into OLCs.

The present study mainly focused on characterizing the cellular properties of OvSCs and Oct4 transfected OvSCs (Oct4-OvSCs) by evaluating the alkaline phosphatase (AP) activity, cell cycle status and cell proliferation as well as expression of cell surface markers and pluripotent transcriptional markers. OvSCs and Oct4-OvSCs differentiated into OLCs were further examined for their ability to initiate and undergo oogenesis in vitro by assessing the morphological characteristics, expression of germ cell specific markers, and measuring the number and size of OLCs. Finally, in vivo folliculogenesis was evaluated by transplanting OvSCs and Oct4-OvSCs into infertile mice.

## Methods

### Chemicals and media

All chemicals and media were purchased from Sigma (St. Louis, MO, USA) and Gibco (Life Technologies, Burlington, ON, Canada), respectively, unless otherwise specified. Media employed for washing was Dulbecco’s phosphate buffered saline (D-PBS) supplemented with 1 mg/ml poly vinyl alcohol (PVA), 1 % penicillin-streptomycin (10,000 IU and 10,000 μg/ml, respectively, Pen-Strep). Advanced Dulbecco’s modified Eagle’s medium (A-DMEM) supplemented with 10 % fetal bovine serum (FBS), 2 mM glutamine and 1 % Pen-Strep was used for cell culture. The pH and osmolality of all media were adjusted to 7.2 and 285 ± 5 mOsm/L, respectively.

### Isolation and culture of ovarian stem/stromal cells (OvSCs)

All experiments were authorized by the Animal Center for Biomedical Experimentation at Gyeongsang National University. The two types of cells [i.e., OvSCs and adult fibroblasts (AFs)] were established from sexually mature 6-month-old female pigs. To isolate OvSCs, ovarian cortex was separated using fine forceps and blade under a microscope, minced into small pieces and then digested by incubating in DPBS containing 1 mg/ml collagenase type I for 1 h. Isolated cells were passed through 100 and 40 μm cell strainer (BD Falcon, MA, USA) sequentially to harvest single cell suspension and washed twice with D-PBS by centrifugation at 500 × g for 5 min. AFs were isolated from ear skin tissues. All the cells were cultured in A-DMEM supplemented with 10 % FBS at 38.5 °C in humidified atmosphere of 5 % CO_2_ in air. Once confluence reached, ovarian stem/stromal cells (OvSCs) were trypsinized using 0.25 % trypsin-ethylenediaminetetraacetic acid (EDTA) solution and pelleted by centrifugation at 500 × g for 5 min. Cells were replated and passaged 4 times for further experiments.

### Transfection of exogenous Oct4 into OvSCs

Oct4 transfection was followed as per the recommended protocol (Allele biotechnology, CA, USA) with minor modifications. Briefly, a total of 1 × 10^5^ OvSCs were transfected with 20 MOI hOct3/4-RFP (O^R^) (ABP-SC-LVIOCT3R, Allele) (Oct4-OvSCs) and polybrene (final concentration, 4 μg/ml) in 2 ml A-DMEM supplemented with 10 % FBS by incubating at 38.5 °C in a humidified atmosphere of 5 % CO_2_ in air. Media was changed 10 h after transfection. OvSCs were also transfected by GFP as a lentiviral infection control. GFP construct was provided by hOct3/4-RFP (O^R^) kit and the transfection method is same as hOct3/4-RFP (O^R^) transfection.

### Alkaline phosphatase (AP) activity

To detect AP activity, the OvSCs attached to the plastic culture surface were fixed with 3.7 % formaldehyde for 1 h. and washed three times with Tris-HCl buffer [100 mM Tris-HCl (Trizma® hydrochloride), 50 mM NaCl, 50 mM MgCl2 · 6H2O and 0.1 % Tween-20]. The cells were stained with AP chromogen kit (BCIP/NBT) (Promega, WI, USA) for 30 min at room temperature. After being washed twice with deionized water, the cells were evaluated for positive activity under a microscope. AFs were used as a negative control.

### Cell cycle, cell proliferation and cell surface markers

The cells at passage 4 with approximately 80 % confluence were analyzed for the stage of cell cycle by fluorescence-activated cell sorting (FACS, Becton Dickinson, NJ, USA). Briefly, after fixation with 70 % ethanol solution at 4 °C overnight, the cells were washed with D-PBS twice and stained with 10 μg/ml propidium iodide in the presence of 2 mg/ml RNase A (Qiagen, Hilden, Germany) for 1 h. at 4 °C. A total of 1 × 10^4^ cells were analyzed for the DNA content in triplicates by flow cytometer and the DNA content of each cells was categorized as G0/G1, S or G2/M stage of the cell cycle.

For analysis of cell proliferation, cells were seeded at 1 × 10^3^ cells/well in a 12-well tissue culture dish in A-DMEM supplemented with 10 % FBS for 14 days. Total cell number of each well was counted with a hematocytometer at every 2 days interval.

Cells were analyzed for the expression of CD markers using flow cytometer. The cells at ~80 % confluence were harvested with 0.25 % trypsin EDTA treatment and blocked with 3 % bovine serum albumin (BSA) solution for 1 h. at 4 °C. For evaluation of CD29 expression, cells were incubated in primary antibody (Mouse anti-pig CD29, 5 μg/ml, BD Pharmingen™, CA, USA) for 1 h. at 4 °C. The cells were washed twice using D-PBS and incubated with fluorescein isothiocyanate (FITC)-conjugated secondary antibody (FITC goat anti-mouse IgG, 5 μg/ml, BD Pharmingen™) for 1 h. at 4 °C. For analysis of CD44, 45 and 90, cells were incubated with FITC-conjugated anti-CD44, 90 (5 μg/ml, BD Pharmingen™) and CD45 (2 μg/ml, Sigma, MO, USA) for 1 h. at 4 °C. The standard was established by appropriate isotype-matched negative controls. FITC–labeled cells were analyzed after acquisition of 1 × 10^5^ events by FACS caliber and Cell Quest software (Becton Dickinson). All experiments were performed in triplicates in three independent experiments.

### Immunofluorescence staining

Cells were fixed with 3.7 % formaldehyde solution for 1 h. at room temperature. After being washed twice with D-PBS, the cells were permeabilized with 0.1 % triton X-100 for 30 min and blocked in D-PBS supplemented with 1 % BSA solution, followed by overnight incubation at 4 °C with primary antibodies against Oct3/4 (Goat polyclonal, 1:200, Santa Cruz Biotechnology, Inc., CA, USA), Nanog (Goat polyclonal, 1:200, Santa Cruz Biotechnology), Sox2 (Rabbit polyclonal, 1:200, Santa Cruz), VASA (Rabbit polyclonal, 1: 100, Abcam, Cambridge, UK) and DAZL (Rabbit polyclonal, 1:100, Abcam) diluted in blocking solution. Slides were rinsed with D-PBS, and then incubated with FITC-conjugated donkey anti-goat IgG or goat anti-rabbit IgG (1:200, Jackson IR laboratories, Inc., PA, USA) for 45 min at 38.5 °C. Slides were then counterstaining with 1 μg/ml 4′,6-diamidino-2-phenylindole (DAPI) for 5 min at room temperature, mounted with Vectashield® (Vector Laboratories, Inc., CA, USA) and observed under a fluorescence microscope (Leica, Wetzlar, Germany).

### Reverse transcription-polymerase chain reaction (RT-PCR)

The expressions of pluripotent transcriptional factors and lineage specific markers were analyzed by RT-PCR. Total RNA was extracted from the cells using RNeasy® mini kit (Qiagen, CA, USA) following the manufacturer’s protocol. cDNA synthesis was performed for 30 min at 55 °C using Omniscript reverse transcription Kit (Qiagen, CA, USA) using oligo-dT primer. PCR was performed using Maxime PCR Premix (iNtRON Biotechnology, Seongnam, Korea) supplemented with each primers and cDNA samples under the following conditions; pre-denaturation at 95 °C for 10 min, denaturation at 94 °C for 30 s, annealing at 56–64 °C for 30 s, elongation at 72 °C for 45 s and final extension at 72 °C for 10 min for 30 cycles using a Eppendorf Mastercycler (Eppendorf, Hamburg, Germany). PCR products were loaded on 1 % agarose gel supplemented with 1 μg/ml ethidium bromide solution for electrophoresis. The results were analyzed by zoom browser EX5.7 software (Primetech, Korea). To quantify the gene expression levels, semi-quantitative RT-PCR was performed, using β-actin for normalization as a reference gene. The gene expression levels were presented as a fold induction with mean ± standard error (SE). The intensities of each band were measured by Gel viewer 1.5 (CURVEX Corp., DAIHAN, South Korea) and calculated against a base of β-actin intensity for relative quantitative analysis. Experiments were performed in triplicates in three independent experiments. The primers used for the study are presented in Table 1. Porcine Oct4 primer (Table 1) was not matched with any sequence in human Oct4 mRNA sequence (GenBank: NM_001285986.1) and it has only matched with porcine Oct4 mRNA Sequence (GenBank: KJ023671.1).

**Table 1:** RT-PCR primers specific to porcine MSCs and differentiated cells

Gene	Primer sequence (5′-3′)	Product Size (bp)	Annealing Tm (°C)	Reference/Accession number
OCT4	F-AGGTGTTCAGCCAAACGACC	335	60	Carlin et al. [12]
	R-TGATCGTTTGCCCTTCTGGC			
SOX2	F-GCCTGGGCGCCGAGTGGA	443	64	Carlin et al. [12]
	R-GGGCGAGCCGTTCATGTAGGTCTG			
Nanog	F-ATCCAGCTTGTCCCCAAAG	438	60	Carlin et al. [12]
	R-ATTTCATTCGCTGGTTCTGG			
GDF9b	F-GGATCCAGAAAAGCACAACC	227	56	Dyce et al. [5]
	R-AGTGTCCAGGGATGAAATGC			
FSHR	F-CTCACCTACCCCAGCCACT	243	56	Dyce et al. [5]
	R-CTCAGGGGAGCAAGTCACAT			
C-MOS	F-AAATCAGCGACTTTGGTTGC	200	56	Dyce et al. [5]
	R-CTGACGCTCCCCTGAGTAAG			
VASA	F-TTGCAGGACGAGATTTGATG	165	56	Dyce et al. [5]
	R-CCAATTCTCGAGTTGGTGC			
ZPC	F-TGGTGTACAGCACCTTCCTG	202	56	Dyce et al. [5]
	R-ATCAGGCGCAGAGAGAACAC			
Stella	F-TTAATCCAACCCGGACTCAG	173	56	Linher et al. [7]
	R-TGGTTGAGGTGGATATTCTGG			
C-kit	F-TGTATTCACAGAGACTTGGCGG	124	56	Linher et al. [7]
	R-CGTTTCCTTTGACCACGTAA			
Β-Actin	F-CTCGATCATGAAGTGCGACG	114	58	SSU07786
	R-GTGATCTCCTTCTGCATCCTGTC			

### Western blot analysis

The expression of Oct3/4, Sox2 and Nanog proteins were evaluated by western blot analysis. Total protein was extracted with RIPA buffer (PIERCE, IL, USA) containing protease inhibitor, quantified using BCA protein assay kit (PIERCE, IL, USA). A total of 20 μg proteins was separated on SDS-PAGE for 60 min at 15 mA per plate and wet transferred electrically onto a nitrocellulose (NC) membrane (Millipore, Eschborn, Germany) for 45 min at 200 mA. NC membrane was blocked with 5 % skim milk in 0.1 % TBST (1 M Tris [pH 7.5], 5 N NaCl and 0.1 % Tween-20) for 1 h. at room temperature. After washing 3 times with 0.1 % TBST, the NC membrane was incubated with 1:200 dilution of Oct3/4, Sox2 and Nanog (Santa Cruz Biotechnology, CA, USA) and 1:1000 dilution of β-actin (Cell Signaling Technology, MA, USA) antibody in 0.1 % TBST – 5 % skim milk for overnight at 4 °C. After being washed 3 times with 0.1 % TBST, the NC membrane was incubated with 1:5,000 dilution of goat conjugate anti-rabbit IgG-HRP or donkey conjugate anti-goat IgG-HRP (Santa Cruz Biotechnology, CA, USA) in 0.1 % TBST – 5 % skim milk for 1 h. at room temperature. Immunoreactivity was detected by enhanced chemiluminescence (ECL; Supersignal® West Pico Chemiluminescent substrate, PIERCE), and then exposed to X-ray film (FUJI Photo Film Co., Ltd, Tokyo, Japan). To quantify the protein expression levels, semi-quantification was performed. The protein expression levels were normalized to β-actin protein levels and presented as a fold induction with mean ± S.E. Experiments were performed in triplicates in three independent experiments. Relative intensities of each band were calculated on the basis of β-actin intensity by Gel viewer 1.5 (CURVEX Corp., DAIHAN, South Korea).

### Differentiation into oocyte-like cells (OLCs) and measuring the number of OLCs

OvSCs and Oct4-OvSCs were differentiated into OLCs following a previously published protocol [5] with minor modifications. Media for induction into OLCs consisted of DMEM and nutrient mixture F12 (DMEM:F12 = 1:1, v/v) supplemented with 5 % FBS, 5 % porcine follicular fluid, 0.23 mM sodium pyruvate, 0.1 mM non-essential amino acids, 2 mM L-glutamine and 0.1 mM β-mercaptoethanol. The cells were cultured in differentiation media for 45 days by changing the media twice a week.

Induced OLCs were confirmed by observing their morphology with increasing size to approximately 50 μm in diameter at day 15, 30 and 45 of differentiation. On day 45, OLCs were stained with 1 μg/ml DAPI and observed under a fluorescence microscope (Nikon, Tokyo, Japan). The expression of pluripotent transcriptional factor, *Oct4*, and germ cell specific markers such as growth differentiation factor 9b (*GDF9b*), *C-Mos*, *Vasa*, *ZPC*, *Stella*, *c-kit* and folliculogenesis marker, *FSHR* was analyzed by using RT-PCR. *β-actin* was used as a control gene. Further, the expressions of Oct3/4, Vasa and DAZL in OLCs were analyzed by immunocytochemistry. In vitro produced porcine mature oocytes were used for positive control for immunocytochemistry.

For evaluating the number of OLCs, cells were seeded at 1 × 10^5^ cells/well in a 24-well plate and differentiated into OLCs for 45 days. On day 45, the floating cells in each well were counted and measured the diameter of the cells using ocular micrometer (Nikon TE300, Japan). The OLCs were classified on their diameter into < 50 μm and > 50 μm in diameter, and if OLCs had zona pellucida, the measurements included its diameter. Experiments were performed in eight replicates in three independent experiments.

### Animal preparation and cell transplantation

Before the cell transplantation, the cells were labeled with fluorescent lipophilic carbocyanine dye PKH26 according to the manufacturer’s instructions (Sigma, MO, USA). The labeled cells were then used for transplantation experiments.

Female BALB/C Nude mice (normal mice), aged 5–6 weeks, weighing approximately 18–20 g, were used in this study. To induce infertility, recipients were sterilized by intraperitoneal injection of busulphan (20 mg/kg, suspended in DMSO), followed by a booster injection after 2 weeks. After 2 weeks from the booster injection, the animals were divided into five groups: control (n = 5, not injected with PBS or cells), PBS injection (n = 5), AFs injection (n = 7), OvSCs injection (n = 10), and Oct4-OvSCs injection (n = 10). After being anesthetized with a combination of 1 μl/g (60 μg/g) Zolazepam/tiletamine (zoletil50, Verbac, France) and 0.5 μl/g Zylazine, mice were injected with 5 μl PBS alone or with 5 μl (1 × 10^4^ cells) of cell suspensions into ovarian cortex using glass pipettes with a 70 μm diameter. Injections of PBS or AFs were evaluated for negative controls in normal and infertile mice.

### Histological assessment and hormone measurement

Sera collected from the mice at 4 weeks after PBS or cell injection was used to measure the estradiol 17β and FSH level using ELISA. Estradiol 17β and FSH were analyzed using enzyme immunoassay kit for estradiol (Oxford Biomedical Research, MI, USA) and FSH (Endocrine Technology, CA, USA) according to the manufacture’s protocol, respectively. Five mice were used for each group and all serum samples and standards were run in duplicate.

The mice were sacrificed at 4 weeks after PBS or cell injection, and ovaries were collected for histological assessment. The ovaries were fixed with 4 % paraformaldehyde for overnight. After being washed with D-PBS 3 times each for 5 min, the ovaries were dehydrated overnight with 20 % sucrose. The dehydrated ovaries were embedded in optical cutting temperature (OCT) compounds (Tissue-Tek®, CE, USA) and sectioned into 8 μm thickness and mounted onto slides. The slides were stained with hematoxylin and eosin (H&E) staining or 1 μg/ml DAPI for 5 min. Images were observed using optical microscope (Nikon TE300, Japan) or fluorescence microscope (Leica CTR600, Switzerland).

For immunohistochemistry, the rabbit specific horseradish peroxidase-diaminobenzidine (HRP-DAB) detection immunohistochemical kit (Abcam) was used. Briefly, sections were incubated with a hydrogen peroxide block solution for 10 min, followed by treating protein block solution for 30 min. After being washed by D-PBS, sections were incubated with the primary antibody, anti-estrogen receptor alpha (rabbit polyclonal, 1:100, Abcam) at 4 °C overnight. Biotinylated goat anti rabbit IgG (H + L) was treated to section for 10 min as a secondary antibody. Visualization was detected using HRP-DAB detection IHC kit according to the manufacturer’s instructions. After counterstaining with hematoxylin, the sections were mounted and observed under the microscope.

### Statistical analysis

Differences among proportional data were analyzed by SPSS 21.0 (SPSS Inc. Chicago, IL. USA). All data was expressed as means ± SE. Comparisons of mean values among groups were performed using student T- test or ANOVA with Tukey’s or Duncan’s multiple comparisons test. Differences were considered to be significant at *P* < 0.05.

## Results

### Increased AP activity in Oct4-OvSCs

**Fig. 1:**
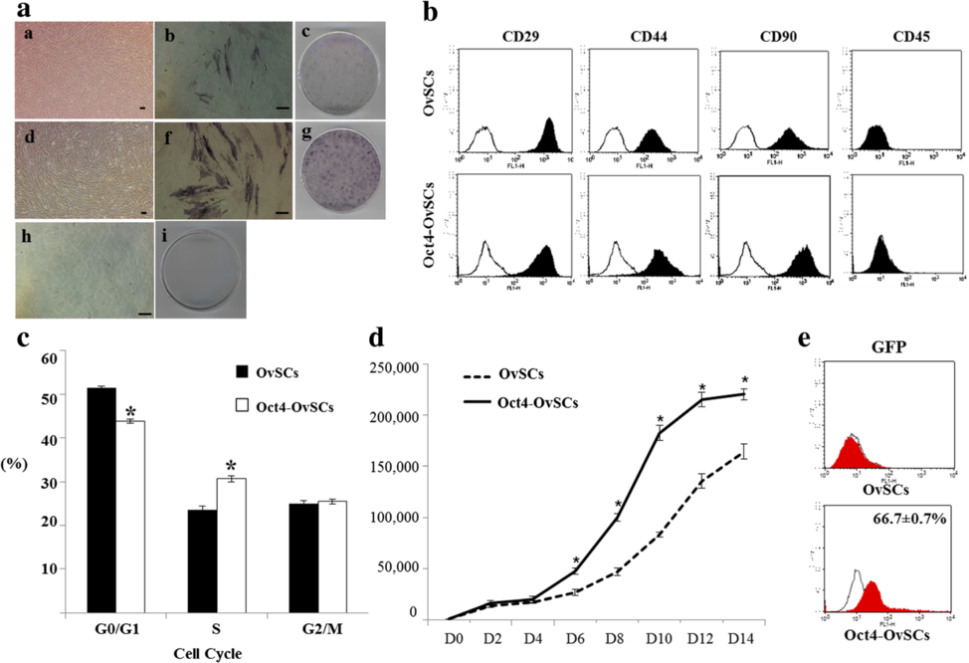
Morphology, AP activity, cell surface markers expression, cell cycle and proliferation of OvSCs and Oct4-OvSCs. **a** The morphology and AP activity of OvSCs (**a** *a*-**a** *c*), Oct4-OvSCs (**a** *d*-**a** *g*) and AFs (**a** *h* and **a** *i*). Purple brown color indicates the positive reaction of AP activity in OvSCs and Oct4-OvSCs. AFs showed no AP activity. Scale bar = 50 μm. **b** Expression of cell surface markers, CD29^+^, CD44^+^, CD90^+^ and CD45^-^. Each sample was counted with 1 × 10^4^ cells by flow cytometer. **c** Cell cycle status, each sample was counted with 1 × 10^4^ cells by flow cytometer (*p < 0.05). **d** Cell proliferation at various time intervals (*p < 0.05). **e** Expression of GFP as a lentiviral infection control analyzed by flow cytometer in Oct4-OvSCs. OvSCs showed no GFP expression (Independent triplicates, Mean ± S.E.)

In primary culture, the OvSCs were grown as heterogeneous populations with elongated, sphere-like or spindle-like adherent features. However, from passage 2–3, the cells became a homogeneous population with a typical fibroblast-like morphology (Fig. 1Aa). After Oct4 transfection of OvSCs, Oct4-OvSCs were also grown into homogeneous populations with a typical fibroblast-like morphology at passage 4 (Fig. 1Ad). OvSCs were transfected with lentiviral vector which expressed GFP as an infection control, and the infection rate in Oct4-OvSCs was found to be 66.7 ± 0.7 %, while non-transfected cells were not detected for GFP (Fig. 1e). At passage 4, OvSCs (Fig. 1Ab and Ac) and Oct-OvSCs (Fig. 1Af and Ag) were positive for AP activity and more population were observed for AP positivity in Oct4-OvSCs compared to OvSCs. These results indicated the existence of stem cells in ovarian cortex and proved the potential stemness in OvSCs transfected with exogenous Oct4. AFs were observed to be negative for AP activity (Fig. 1Ah and Ai).

### Expression of mesenchymal cell surface markers in OvSCs and Oct4-OvSCs

The expression of mesenchymal cell surface specific markers, such as CD29, CD44 and CD90 were analyzed in OvSCs and Oct4-OvSCs using flow cytometer at passage 4 (Fig. 1b). The results were presented as percentage mean positive cells of surface markers in analyzed cell population. The percentage of cells that expressed CD44 was significantly (*P* <0.05) greater in OvSCs than that in Oct4-OvSCs (98.7 ± 0.7 % vs. 94.7 ± 0.3 %). CD29 and CD90 were highly expressed in both OvSCs and Oct4-OvSCs with no significant differences. However, the hematopoietic cell lineage marker, CD45, was not detected in OvSCs and Oct4-OvSCs (Fig. 1b).

### Enhanced cell proliferation in Oct4-OvSCs

Flow cytometer analysis showed that the population of G0/G1 phase of the cell cycle in Oct4-OvSCs was significantly (*P* <0.05) lower than OvSCs (43.8 ± 0.5 % vs. 51.5 ± 0.3 %) (Fig. 1c). In contrast, the population of S phase of the cell cycle in Oct4-OvSCs was significantly (*P* <0.05) higher than OvSCs (30.7 ± 0.7 % vs. 23.6 ± 0.8 %). These results indicated that the Oct4 transfection resulted in enhanced cell proliferation. The population of cells at G2/M phase in Oct4 transfected cells and OvSCs was observed to be 25.5 ± 0.6 and 24.9 ± 0.7 respectively.

Further, to confirm the cell proliferation ability, OvSCs and Oct4-OvSCs were compared for growth kinetics at passage 4 (Fig. 1d). Growth pattern of these cells revealed a sigmoid curve, wherein proliferation of Oct4-OvSCs was significantly (*P* <0.05) more rapid than OvSCs from day 6 to 14. There was no significant difference in cell proliferation between OvSCs and Oct4-OvSCs at day 2 and 4 of culture.

### Variation of expression of pluripotency-related transcription factors after Oct4 transfection

**Fig. 2:**
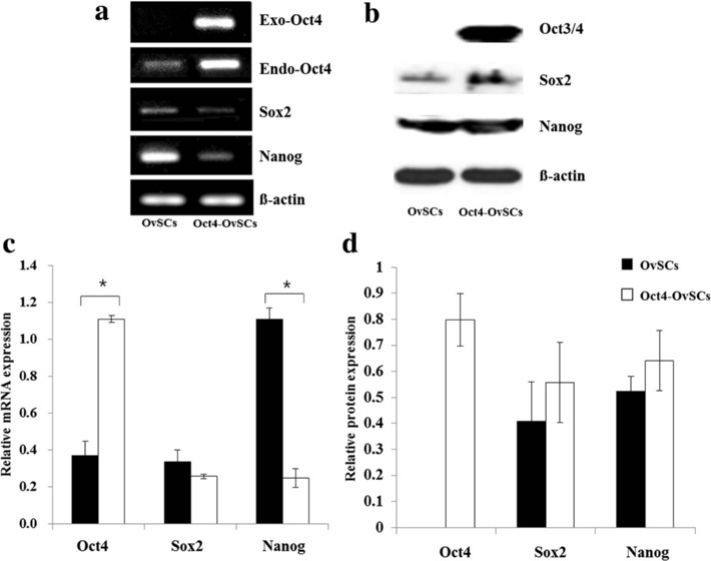
Expression of pluripotency-related transcription factors in OvSCs and Oct4-OvSCs analyzed by RT-PCR and western blot. **a** Result of RT-PCR for exogenous Oct4, endogenous Oct4, Sox2 and Nanog. **b** Western blot analysis for Oct3/4, Sox2 and Nanog. β-actin was used as a reference. **c** Relative mRNA abundance of endogenous Oct4, Sox2 and Nanog with a base of b-actin intensity. **d** Relative protein abundance of Oct3/4, Sox2 and Nanog with a base of β-actin intensity. Three independent experiments performed in triplicates were used for C and D (*p < 0.05)

The expression of pluripotent transcriptional factors, such as Oct3/4, Sox2 and Nanog, in OvSCs and Oct4-OvSCs was analyzed by RT-PCR and western blot analysis (Fig. 2). The exogenous *Oct4*, which was transfected using human Oct3/4 lentiviral particle, was expressed in Oct4-OvSCs, whereas in OvSCs, it was not expressed (Fig. 2a). The endogenous *Oct4* was expressed in OvSCs and Oct4-OvSCs as revealed by RT-PCR (Fig. 2a). However, the intensity of endogenous *Oct4* was significantly (*P* <0.05) higher in Oct4-OvSCs than OvSCs (Fig. 2c). The *Sox2* was also expressed in OvSCs and Oct4-OvSCs, and there was no significant difference between these cells based on band intensities (Fig. 2a and c). *Nanog* was also expressed in OvSCs and Oct4-OvSCs, with significantly (*P* <0.05) higher level in OvSCs than Oct4-OvSCs (Fig. 2a and c). Protein levels of Oct3/4, Sox2 and Nanog were evaluated by western blot analysis. Oct3/4 protein was expressed only in Oct4-OvSCs, but not in OvSCs (Fig. 2b and d). Sox2 and Nanog were detected in OvSCs and Oct4-OvSCs, with no significant difference among these cells (Fig. 2b and d).

### Enhanced OLCs formation and expression of transcriptional and germ cell markers in Oct4-OvSCs

**Fig. 3:**
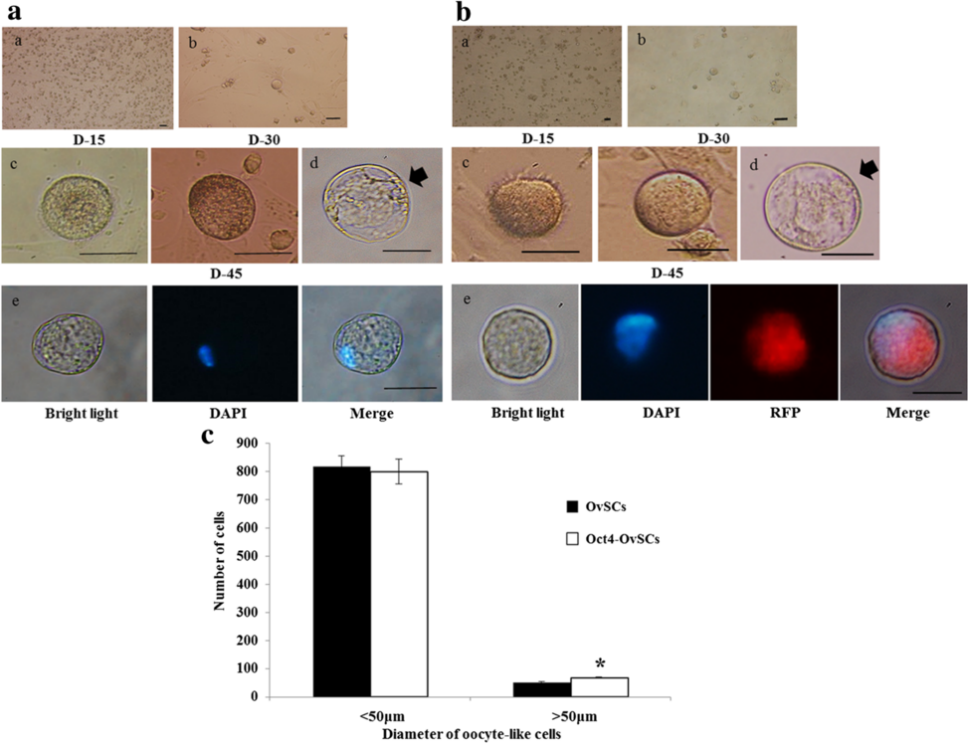
In vitro differentiation of OvSCs and Oct4-OvSCs into oocyte-like cells. **a** and **b** Following the induction, OvSCs (**a** *a* and **a** *b*) and Oct4-OvSCs (**b** *a* and **b** *b*) were changed to distinct sphere-like morphology during the period of 15 to 30 days of induction. At day 45 of differentiation, the cells were changed their morphology to ‘big round-cells’, so called OLCs in OvSCs (**a** *c*-**a** *e*) and Oct4-OvSCs (**b** *c*-**b** *e*). Zona pellucida (Black arrow) was observed in OLCs (**a** *d* and **b** *d*). Nuclear staining by DAPI was observed in OLCs differentiated from OvSCs (**a** *e*) and Oct4-OvSCs (**b** *e*). OLCs derived from Oct4-OvSCs expressed red fluorescence protein through cytoplasm (**b** *e*). Scale bar = 50 μm. **c** The number and diameter of OLCs from OvSCs and Oct4-OvSCs. At day 45 of differentiation, the floating cells were counted and measured the diameter of the cells (Three independent experiments performed in 8 replicates, *p < 0.05)

Before induction into OLCs, OvSCs and Oct4-OvSCs had a fibroblast-like morphology (Fig. 1Aa and Ac). Following the differentiation, the cells changed to a distinct sphere-like morphology during the next 15 to 30 days of culture in the differentiation media (OvSCs, Fig. 3Aa and Ab; Oct4-OvSCs, Fig. 3Ba and Bb). At day-45 of differentiation, the morphology of cells was changed to ‘big round-cells’, which had approximately 50 μm in diameter, so called oocyte-like cells (OLCs) (Fig. 3Ac and Bc), which characteristically possessed zona pellucida like structure. Further, nuclear staining by DAPI was observed in OLCs derived from OvSCs (Fig. 3Ad and Ae) and Oct4-OvSCs (Fig. 3Bd and Ae) under a fluorescent microscope. OLCs derived from Oct4-OvSCs expressed a cytoplasmic red fluorescence protein (Fig. 3Be).

The number and diameter of OLCs derived from Oct4-OvSCs and OvSCs was analyzed by counting the floating cells (Fig. 3c). At day-45 of differentiation, the floating cells were counted and diameter of the cells was measured. The number of floating cells, less than 50 μm in diameter, was similar between Oct4-OvSCs and OvSCs (800.0 ± 44.3 vs. 818.7 ± 38.9). However, the OLCs from Oct4-OvSCs were generated significantly (*P* <0.05) higher number of cells with > 50 μm in diameter than OvSCs (68.7 ± 1.7 vs. 51.4 ± 2.9).

**Fig. 4:**
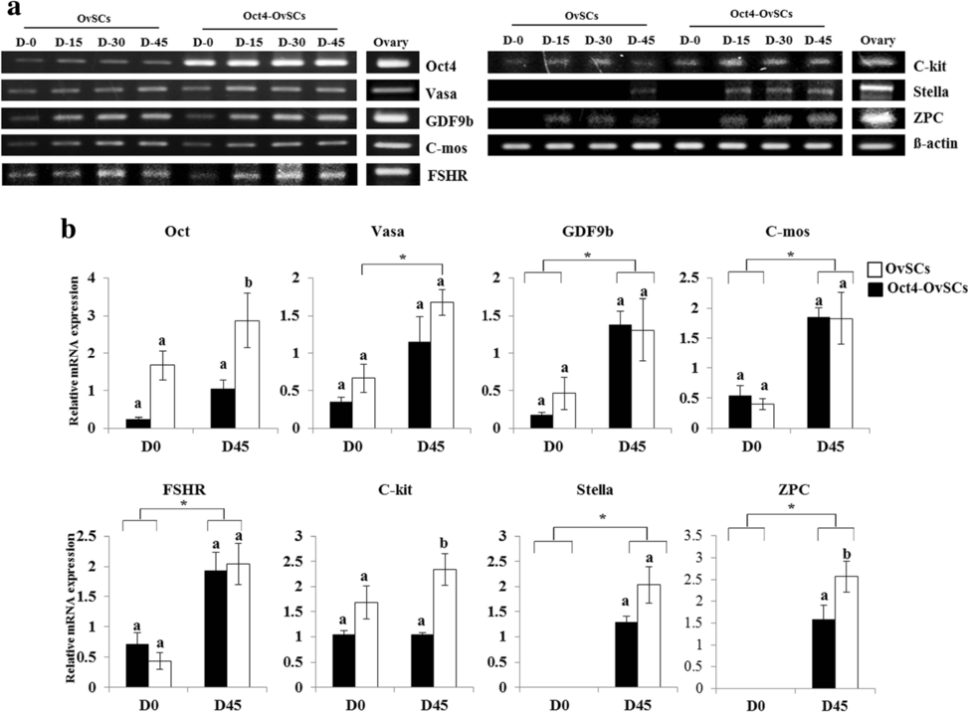
The expression of transcriptional, germ cell specific and folliculogenesis markers following the differentiation into OLCs. **a** These markers were analyzed on day 0, 15, 30 and 45 of differentiation. β-actin was used as a reference gene. Porcine ovarian tissue was used for positive control. **b** Relative mRNA abundance of Oct4, Vasa, GDF9b, C-Mos, FSHR, C-kit, Stella and ZPC with a base of β-actin intensity. Different superscript letters represent a significant difference between OvSCs and Oct4-OvSC on day 0 or 45 of differentiation. Asterisk indicates a significant difference between the day 0 and 45 of differentiation in OvSCs or Oct4-OvSCs. Three independent experiments performed in triplicates were used and multiple comparisons were performed by Duncan test (Mean ± S.E, *p* < 0.05)

**Fig. 5:**
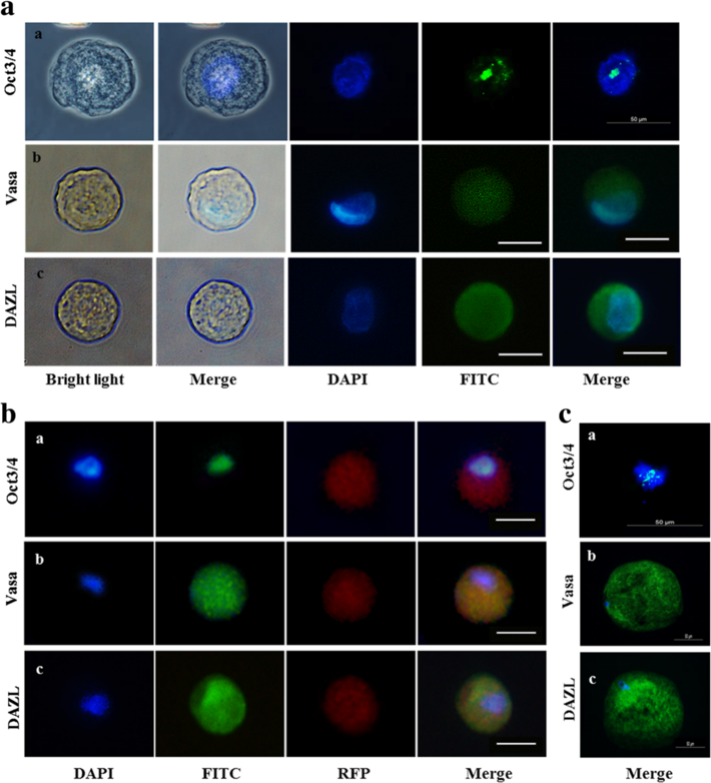
Characterization of OLCs derived from OvSCs and Oct4-OvSCs by immunocytochemistry. **a** and **b** Expression of Oct3/4, VASA and DAZL were detected in OLCs from OvSCs and Oct4-OvSCs, respectively. Green fluorescence (FITC) indicates positive reaction and blue fluorescence indicates nuclei (DAPI). Red fluorescence (RFP) indicates transfection of exogenous Oct4. **c** Expression of Oct3/4, VASA and DAZL in natural oocytes as a positive control. Scale bar = 50 μm

To characterize the differentiated cells into OLCs from OvSCs and Oct4-OvSCs, the expression of transcription factor (*Oct4*), germ cell and meiosis specific markers (*Vasa*, *GDF9b*, *C-mos*, *C-kit*, *Stella* and *ZPC*), and folliculogenesis marker (*FSHR*) was evaluated at mRNA level using RT-PCR. The relative expression levels were quantified using band intensities (Fig. 4a and b). Ovarian tissues were used as positive control and they expressed all oocyte specific markers (Fig. 4a). *Oct4*, *Vasa*, *GDF9b*, *C-mos*, *FSHR* and *C-kit* were detected in OvSCs and Oct4-OvSCs at all time points during the differentiation that were examined (Fig. 4a). Overexpression of *Oct4* was displayed with higher level of *Oct4* at each point till day 45 of differentiation of Oct4-OvSCs than OvSCs based on band intensities. However, the expression level of *Oct4* did not differ between day 0 and 45 of differentiation in both OvSCs and Oct4-OvSCs (Fig. 4b). The expression of *Vasa* was significantly (*P* <0.05) increased in Oct4-OvSCs after differentiation into OLCs, but not in OvSCs (Fig. 4b). Further, there was no significant difference in the expression of *Vasa* among OvSCs and Oct4-OvSCs on day 45 of differentiation. *GDF9b*, *C-mos* and *FSHR* were expressed significantly (*P* <0.05) higher in both OvSCs and Oct4-OvSCs on day 45 of differentiation (Fig. 4b), although differences between cell types were not significant. *C-kit* level was significantly (*P* <0.05) higher in Oct4-OvSCs than OvSCs on day 45 of differentiation, but there was no significant difference between day 0 and 45 of differentiation (Fig. 4b). The expression of *Stella* initiated on day 45 and 15 of differentiation in OvSCs and Oct4-OvSCs, respectively (Fig. 4a). However, there was no significant difference in relative mRNA level of *Stella* between OvSCs and Oct4-OvSCs on day 45 of differentiation (Fig. 4b). The expression of *ZPC* initiated on day 15 of differentiation in both OvSCs and Oct4-OvSCs, and its expression level was found to be significantly (*P* <0.05) higher in Oct4 OvSCs compared to OvSCs on day 45 of differentiation (Fig. 4a and b). OLCs derived from OvSCs and Oct4-OvSCs were further analyzed by immunocytochemistry to detect the expression of Oct3/4, Vasa and DAZL at protein level (Fig. 5). Oct3/4 was expressed in the nucleus of OLCs, whereas Vasa and DAZL were detected in the cytoplasm (Fig. 5a and b) and these expression patterns were similar to in vitro matured oocytes (Fig. 5c).

### Follicle formation in OvSCs and Oct4-OvSCs transplanted mice

**Fig. 6:**
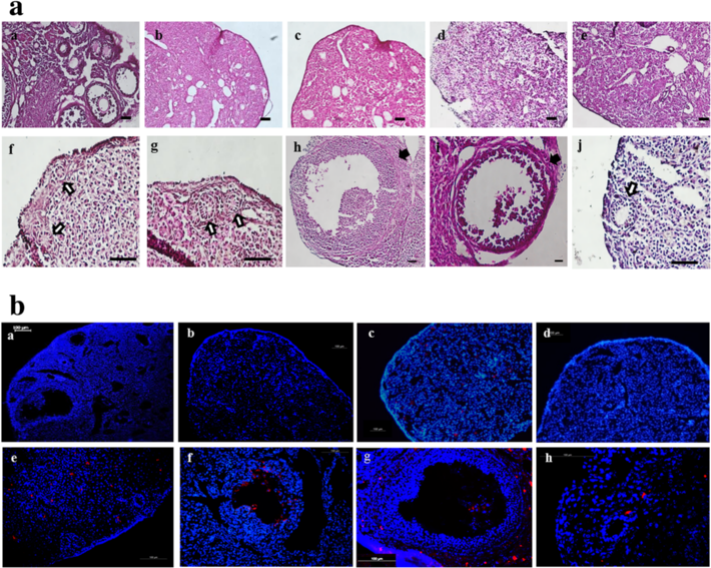
Histological observation of mouse ovaries following cell transplantation into infertile mouse. **a** Histological observation by H&E staining. Image of normal folliculogenesis in BALB/C nude mice ovary (**a** *a*). After twice busulphan injection at 2 weeks interval, follicles and germ cells were destroyed completely in ovaries when observed at 2 and 6 weeks after the second injection (**a** *b* and **a** *c*, respectively). Cells or PBS injection was performed at 2 weeks after the second busulphan injection. Injection of AFs or PBS into sterile ovaries (**a** *d* and **a** *e*, respectively). Transplantation of OvSCs (**a** *f* and **a** *g*) and Oct4-OvSCs (**a** *h*-**a** *j*). White arrows indicate new primary follicles while black arrows indicate a developing follicle containing theca and groanulosa cells. **b** Histological observation by DAPI staining. Image of normal folliculogenesis (**b** *a*). Sterile mouse ovary at 6 weeks after the second busulphan injection (**b** *b*). Ovaries injected AFs or PBS (**b** *c* and **b** *d*, respectively). Transplantation of OvSCs (**b** *e*) and Oct4-OvSCs (**b** *f*-**b** *h*). Red fluorescence indicates PKH26 labeled cells and blue florescence indicates nucleus. Scale bar = 100 μm

Control BALB/C nude mice ovary revealed intact follicles containing oocytes and were found to be progressing through folliculogenesis (Fig. 6Aa and Ba). Upon injecting busulphan twice at 2 week intervals resulted in a disruption of folliculogenesis and destruction of germ cells including oocytes in ovaries when observed at 2 and 6 weeks after the second injection (Fig. 6Ab and Ac). A total of 1 × 10^4^ cells were transplanted into ovaries of busulphan treated mice. At 4 weeks after OvSCs transplantation into sterile ovaries, primary follicles were observed in the ovaries (Fig. 6Af and Ag). Furthermore, the ovaries transplanted with Oct4-OvSCs showed not only primary follicles (Fig. 6Ai) but also developing follicles containing theca and granulosa cells (Fig. 6Ah and Ai). Under the fluorescence microscopy, Oct4-OvSCs labeled with PKH26 were observed in developing follicles (Fig. 6Bf and Bg). However, OvSCs and Oct4-OvSCs labeled with PKH26 were not located in primary follicles (Fig. 6Be and Bh). The labeled AFs did not induce any follicles in sterile ovaries (Fig. 6Ad and Bc). The labeled cells were not observed in PBS injected ovaries (Fig. 6Ae and Bd).

**Fig. 7:**
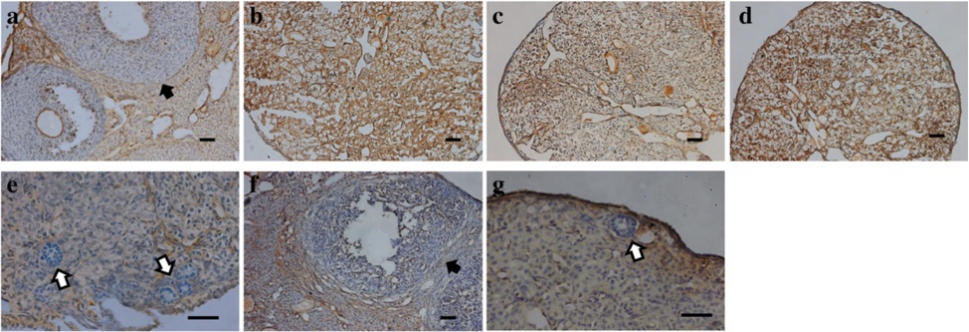
Expression of estrogen receptor alpha (ERα) in mouse ovaries. Brown color indicates the positive expression of ERα. **a** Image of normal mouse ovaries. **b** Sterile mouse ovary at 6 weeks after the second busulphan injection. **c** and **d** AFs or PBS injected mouse ovaries. **e** OvSCs transplanted ovaries. **f** and **g** Oct4-OvSCs transplanted ovaries. White arrow indicates the primary follicle which was not expressed ERα. Black arrow means ERα expressed in theca cells. Scale bar = 100 μm

Estrogen receptor alpha (ERα) was expressed in theca cells and interstitial cells in normal ovaries that were not injected with busulphan (Fig. 7a). After injection of busulphan in mouse, ERα was expressed in all interstitial cells in sterile ovaries (Fig. 7b). AFs or PBS injected ovaries also expressed ERα in all interstitial cells in infertile mice (Fig. 7c and d). In OvSCs transplanted ovaries, ERα was not expressed in primary follicles containing granulosa cells, but expressed in interstitial cells (Fig. 7e). ERα was expressed in theca cells of developing follicles and interstitial cells, but not in primary follicles in Oct4-OvSCs transplanted ovaries (Fig. 7f and g).

### Normal estradiol and FSH level in OvSCs and Oct4-OvSCs transplanted mice

**Fig. 8:**
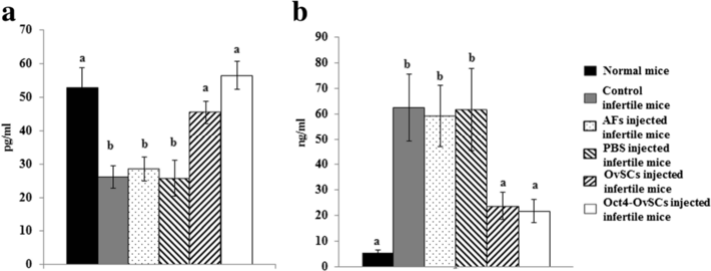
Hormone (Estrogen and FSH) concentration in mouse serum. **a** and **b** Mouse serum was collected and the concentration of estradiol and FSH were measured by ELISA kit. Different superscript letters represent a significant difference among the groups. Three independent experiments performed in five replicates were used and multiple comparisons were performed by Tukeys test (Mean ± S.E, *p* < 0.05)

To evaluate the concentration of estradiol and FSH in mouse serum, ELISA analysis was performed (Fig. 8a and b, respectively). Control infertile mice revealed significantly (*P* <0.05) lower estradiol concentration than normal female BALB/C Nude mice (26.0 ± 3.3 vs. 52.7 ± 6.1 pg/ml), and showed similar estradiol concentration to AFs or PBS injected infertile mice (28.5 ± 3.6 and 25.75 ± 5.4 pg/ml). However, OvSCs or Oct4-OvSCs transplantation resulted in higher estradiol concentration in infertile mice. The concentrations of estradiol in OvSCs injected mice (45.5 ± 3.3 pg/ml) were lower than normal and Oct4-OvSCs injected mice (56.5 ± 4.3 pg/ml), but there was no significant difference among these groups.

FSH concentration of control infertile mice was significantly (*P* <0.05) higher than normal mice (62.5 ± 13.2 vs. 5.1 ± 0.9 ng/ml). After injection of AFs or PBS into infertile mice, FSH concentration was still significantly (*P* <0.05) higher than normal mice (59.1 ± 12.0 and 61.6 ± 16.3 vs. 5.1 ± 0.9 ng/ml). However, FSH concentration decreased by transplantation of OvSCs or Oct4-OvSCs into infertile mice. OvSCs or Oct4-OvSCs injected mice had lower FSH concentration than control infertile mice (23.8 ± 5.4 and 21.8 ± 4.6 vs. 62.5 ± 13.2 ng/ml). The FSH concentration of OvSCs or Oct4-OvSCs was higher than normal mice, but there was no significant difference.

## Discussion

Ovarian stem cells, commonly isolated from ovarian surface epithelium or ovarian germinal epithelium [15], are also found in ovarian cortex enclosed by epithelial like cells of irregular shape. Recent study has reported two different types of stem cells in the ovary named as ovarian germ stem cells (OGSCs) and very small embryonic-like stem cells (VSELs) [23]. OvSCs used in this study showed fibroblast like morphology with a size of 10–15 μm. Therefore, OvSCs in the present study are comparable with OGSCs (size >8 μm) than VSEL (3–5 μm). Further, VSEL expresses nuclear OCT4, while OGSE expresses cytoplasmic OCT4 at protein level, suggesting VSELs are more pluripotent. In the present study OvSCs did not express Oct3/4 protein suggesting their less pluripotent status. Therefore, in our view, OvSCs used in this study were more similar to OGSCs in terms of their size and pluripotent status when compare to VSELs. In the present study, we isolated OvSCs from the ovarian cortex and were characterized. Additionally, exogenous Oct4 was transfected to OvSCs to further understand their characteristics and function towards differentiation into OLCs. Both OvSCs and Oct4-OvSCs grew into homogeneous populations with a typical fibroblast-like morphology similar to those of porcine MSCs or MSC-like cells derived from various sources including bone marrow, skin, adipose tissue, umbilical cord matrix and ovarian stroma [9, 10, 24–26]. Oct4-OvSCs displayed more population of AP positive cells compared to non-Oct4 transfected OvSCs, these observations indicated the enhanced stemness in OvSCs upon transfection with exogenous Oct4 [12, 27].

The expression of mesenchymal cell surface markers analyzed using flow cytometer revealed that both OvSCs and Oct4-OvSCs highly expressed CD29, 44 and 90, but negative for hematopoietic cell lineage marker, CD45. The CD marker expression patterns of OvSCs and Oct4-OvSCs were similar with those of porcine MSCs [9, 10, 26].

The capacity of cell proliferation was promoted by Oct4 transfection in human MSCs [28]. In our study, transfection of Oct4 into OvSCs increased the cell population with S phase of the cell cycle, and Oct4-OvSCs had higher proliferation ability than OvSCs. These results are in agreement with previous findings, wherein Oct4 transfected MSCs resulted in higher proliferation ability [28, 29]. Oct4, Sox2 and Nanog are important transcription factors for regulation of pluripotency and maintenance of self-renewal ability in stem cells [21, 30–32]. Recent studies have shown the expression of these transcription factors in adult stem cells of various sources, such as skin, adipose tissue, bone marrow and umbilical cord matrix in porcine [9, 12, 33]. Additionally, Song et al. [9] previously reported that these factors were expressed in OvSCs in newborn miniature pig. We detected the expression of Oct4 in OvSCs at mRNA level with no expression at protein level, suggesting these cells may not have the ability to translate to Oct3/4 protein even though Oct4 mRNA was detected in the cell population. Endogenous Oct4 expression was significantly higher in Oct4-OvSCs at mRNA levels than control OvSCs, and Oct3/4 protein was also detected in Oct4-OvSCs, suggesting that the exogenous Oct4 transfection has induced an increase in endogenous Oct4 at mRNA level and might potentiated the cell’s ability to translate it into Oct4 protein. The previous reports have demonstrated that Oct4 overexpression had the ability to generate induced pluripotent stem cells in mouse and human [28, 34, 35]. The expression levels of pluripotent genes, including Sox2 and Nanog, increased by induction to pluripotent stem cell [28, 35]. Another report [29] has demonstrated that the overexpression of Oct4 in human MSCs derived from the bone marrow at later passage has increased the expression of Nanog. However, the present study showed that the expression levels of Sox2 and Nanog did not increased by Oct4 transfection in OvSCs, even though transfected cells were expressed mesenchymal lineage markers. This contrasting result might be due to the difference in cell source between the experiments.

In vitro differentiation of OvSCs and Oct4-OvSCs into OLCs [5], after 45 days of differentiation, cells changed their morphology to ‘big round cells’, which had approximately 50 μm diameter and cells revealed zona pellucida-like structure with a nucleus. These findings are in agreement with the earlier reports of OLCs induced from various tissue origins [2, 5, 6, 8, 13]. After induction for 45 days, the number of OLCs, less than 50 μm in diameter, did not differ between Oct4-OvSCs and OvSCs, However, OLCs, more than 50 μm in diameter, were more abundant in Oct4-OvSCs when compared to OvSCs. These findings were in accordance with the results of gene expression, in which germ cell specific genes were expressed higher in OLCs from Oct4-OvSCs than those from OvSCs.

To confirm the ability to form OLCs, the expression of transcription factors (Oct4), germ cell and meiosis specific markers (Vasa, GDF9b, C-mos, C-kit, Stella and ZPC), and marker of folliculogenesis (FSHR) was evaluated by RT-PCR and quantified their relative expression using respective band intensities. Oct4, C-kit and ZPC expression was significantly higher in Oct4-OvSCs than OvSCs on day 45 of differentiation, which indicates that Oct4-overexpression resulted in an increase of these gene expressions in differentiated OLCs. The expression of Vasa, GDF9b, C-mos, FSHR, Stella and ZPC was significantly increased after differentiation into OLCs in OvSC or Oct4-OvSCs. Development of OLCs has already been confirmed under different culture conditions with the expression of several oocyte specific genes such as Vasa, DAZL, GDF9b, MLH1*,* SCP1*,* PUM1, PUM2, and POU5F1 [36]. Late stage germ cell markers, such as Vasa and GDF9b, and granulosa cell marker, FSHR, were detected at D0 of differentiation in this study, suggesting a possible germ cell and/or granulosa cell contamination in the isolated OvSCs. However, we exclude this possibility, since 40 μm cell strainer was used to prevent contamination from primary oocytes when OvSCs were isolated. Further, only cortex part was collected under a microscope to avoid the contamination of granulosa and theca cells. Nevertheless, granulosa cells usually need different culture conditions [37, 38] compared to OvSC culture. Therefore, we assume that the granulosa cells should have eliminated during passaging as we used passage 4 cells in our experiment. It has been also reported that ovarian stem cells derived from the ovarian surface epithelium (OSE) expresses GDF-9 and VASA in mouse [39], suggesting ovarian stem cells used in the present study could express germ cell marker before differentiating into OLCs. Previous report also demonstrated the similar results for differentiation of ovarian theca stem/stromal cells into OLCs [40]. To further evaluate the expression of Oct3/4, Vasa and DAZL at protein level, OLCs were analyzed by immunocytochemistry. Oct3/4 was strongly expressed in the nucleus of OLCs, whereas Vasa and DAZL were detected in the cytoplasm. These expression patterns were similar to in vitro matured oocytes and suggested that these OLCs have similar protein expression of germ cell markers to natural oocytes.

In vivo folliculogenesis was observed upon transplantation of OvSCs and Oct4-OvSCs into the infertile mice ovaries. The present study showed that busulphan treatment into BALB/C nude mice completely destroyed the follicles and germ cells in ovaries when observed at 2 and 6 weeks after the second injection [3, 17]. Female mouse germline stem cells were transplanted into infertile mouse ovaries and subsequently produced new oocyte and follicle in vivo [1, 3]. After transplantation of putative theca stem cells into mouse ovary, the cells were observed at the thecal layer of follicles [41]. In the present study, OvSCs and Oct4-OvSCs transplantation into ovaries were clearly contributed to the formation of follicles and its associated cells, such as theca and granulosa cells. OvSCs transplanted into infertile mice resulted in the generation of primary follicles. On the other hand, the ovaries transplanted with Oct4-OvSCs not only generated primary follicles, but also the developing follicles containing theca and granulosa cells. This suggested that Oct4-OvSCs transplantation had greater capacity for folliculogenesis in infertile mice. Further, the developing follicles contained Oct4-OvSCs labeled with PKH26, but primary follicles lacked, suggesting inclusion of Oct4-OvSCs in primary follicles may stimulate the advancement in folliculogenesis. It has been previously reported that granulosa cell nests determine the formation of primordial follicles, as it migrates to the cortex from ovarian stromal layer [42].

Estrogen receptor alpha (ERα) has been shown to be expressed in theca cells and interstitial cells, but not in granulosa cells [43–45]. In the present study, granulosa cells in primary follicles did not expressed ERα in OvSCs or Oct4-OvSCs transplanted ovaries. Further, ERα was expressed in theca cells and interstitial cells in Oct4-OvSCs transplanted ovaries. It was demonstrated that ERα expression in OvSCs or Oct4-OvSCs transplanted ovaries were similar to normal mice ovaries.

Infertile mice generated by busulphan injection have been reported to show lower estradiol and higher FSH concentration in serum than the normal mice [46–48]. OvSCs or Oct4-OvSCs transplantation into infertile mice resulted in similar estradiol and FSH concentration as observed in the normal intact mice. Further, Oct4 overexpression in OvSCs resulted in increased level of estradiol concentration when compare to OvSCs upon transplantation into mice. Recent study showed that administration of ovarian cell-like cells differentiated from postnatal mouse skin cells into ovariectomized mice lead to increase in estradiol level. Although these findings are not exactly similar to our observations, these ovarian cell-like cells have function in vivo for recovery of endocrine disorders [49].

## Conclusions

These findings demonstrated that putative stem cells were present in ovarian cortex and have the differentiation ability into OLCs and folliculogenesis in vivo. Oct4-overexpression enhanced the AP activity, cell proliferation and differentiation into OLCs compared to OvSCs. In addition, the ability of in vivo folliculogenesis and hormone secretion suggested that Oct4-OvSCs could be considered as a useful in vitro cellular model for understanding the mechanisms of oogenesis and folliculogenesis. However, further studies are required to analyze the over-expression or down-regulation of germ cell specific markers in these stem cells to completely understand the mechanisms of folliculogenesis and oogenesis.

### Statement of ethics approval

This study was approved by the Animal Center for Biomedical Experimentation at Gyeongsang National University via GNU-120404-M0006.

### Consent for publication

Not Applicable.

### Availability of data and materials

Not Applicable.

## Abbreviations

GDF9b: growth differentiation factor 9b
OGSCs: ovarian germ stem cells
OvSCs: ovarian stem/stromal cells
VSELs: very small embryonic-like stem cells
